# Double-Camera Fusion System for Animal-Position Awareness in Farming Pens

**DOI:** 10.3390/foods12010084

**Published:** 2022-12-23

**Authors:** Shoujun Huo, Yue Sun, Qinghua Guo, Tao Tan, J. Elizabeth Bolhuis, Piter Bijma, Peter H. N. de With

**Affiliations:** 1 Department of Electrical Engineering, Eindhoven University of Technology, 5612 AP Eindhoven, The Netherlands; 2 Department of Mathematics and Computer Science, Eindhoven University of Technology, 5612 AZ Eindhoven, The Netherlands; 3 Department of Animal Sciences, Wageningen University & Research, 6700 AH Wageningen, The Netherlands

**Keywords:** double-camera, convolutional neural network, fine-tune, image registration

## Abstract

In livestock breeding, continuous and objective monitoring of animals is manually unfeasible due to the large scale of breeding and expensive labour. Computer vision technology can generate accurate and real-time individual animal or animal group information from video surveillance. However, the frequent occlusion between animals and changes in appearance features caused by varying lighting conditions makes single-camera systems less attractive. We propose a double-camera system and image registration algorithms to spatially fuse the information from different viewpoints to solve these issues. This paper presents a deformable learning-based registration framework, where the input image pairs are initially linearly pre-registered. Then, an unsupervised convolutional neural network is employed to fit the mapping from one view to another, using a large number of unlabelled samples for training. The learned parameters are then used in a semi-supervised network and fine-tuned with a small number of manually annotated landmarks. The actual pixel displacement error is introduced as a complement to an image similarity measure. The performance of the proposed fine-tuned method is evaluated on real farming datasets and demonstrates significant improvement in lowering the registration errors than commonly used feature-based and intensity-based methods. This approach also reduces the registration time of an unseen image pair to less than 0.5 s. The proposed method provides a high-quality reference processing step for improving subsequent tasks such as multi-object tracking and behaviour recognition of animals for further analysis.

## 1. Introduction

Animals are one of the primary food sources for society, supplementary to vegetables and cereals [1]. Pigs are one of the most commonly raised livestock in the world, and they are the primary protein supplier for millions of people [2]. The world population is projected to be 9.2 billion in 2050 [3] and food security is facing more severe global challenges. However, traditional pig farms produce many greenhouse gasses and cause water pollution [4], which urgently need to transform into precision livestock farming (PLF) [5]. PLF has resulted in the development of intelligent tools to manage animals. These tools take the precise measurement, monitoring, and tracking of individual and group animals, yielding to automated status and condition estimations of the animals [6,7,8,9]. Nowadays, computer vision technologies are increasingly employed for livestock monitoring [7,8,9]. The application of computer vision to monitor animal farms (e.g., pigs) can address a broad range of monitoring and analysis tasks [7]. Cowton et al. [10] have designed a complete system for recognizing, tracking, and extracting metrics related to individual pigs via RGB cameras. Specifically, they combined a faster region-based convolutional neural network with DeepSORT and used a transfer learning strategy during the training. The authors have evaluated the method on their pig detection dataset and achieved an average precision of 0.901 for recognizing pigs in frames. The result has enabled to yield 92% for Multi-object tracking accuracy (MOTA) and 73.4% for identity F1-score (IDF1). The above-mentioned study is based on image data captured by a single-camera system in a fixed position. In large-scale farming scenarios, higher object density typically causes more occlusions. In addition, different lighting conditions, such as overexposure within a recording view, also generate challenges to subsequent data analysis. In this work, we propose to install two tilted cameras at the top of pig pens with an overlapping field of view. Compared with single-camera-based monitoring, a double-camera-based monitoring system can capture more appearance details of each pig from different viewpoints. However, this raises a challenge for the optimal fusion of the information from each individual camera.

Recently, many researchers have conducted studies on double-camera or multi-camera fusion, where most studies focused on pedestrian and vehicle tracking. In order to improve the detection performance of the multi-camera system under occlusion conditions, Wu et al. [11] have proposed a Multi-View Region Proposal Network (MVRPN) to localize the vehicles on the ground plane. The authors have used multiple side views to evaluate the ground-occupancy vector of the vehicles, where MVRPN is trained under the supervision of top-view ground-plane information. The method achieves an average precision of 0.789 and a multi-object detection precision of 0.708 on a dataset captured on the road in Richardson, TX, USA. Wang et al. [12] have unified information from multiple cameras by transferring object representations from multiple side views to the top view. First, the authors apply a detector to each side view to obtain the detection results. Then they use a neural network to map these results into top-view representations. Finally, the authors have applied a clustering algorithm to the mapped object representations in the top view to fuse the information from multiple cameras. Wang et al. have obtained a distance error of 0.019 in normalized coordinates on their dataset.

These research works are very ingenious at integrating information and performing well in their respective tasks. However, these works require feature engineering or complete detection and trajectory extraction using a single camera prior to information fusion. The single-camera feature engineering inevitably leads to the loss of information. Furthermore, providing the image information from only one camera for the receptive field of a convolutional neural network can potentially lead to under-utilizing the feature characterization. In addition, the use of a single camera has shortcomings in solving occlusions when animals are densely packed and there are quality limitations in the corner views. Therefore, we propose a method to find the spatial correspondence at the pixel level in a double-camera system. In this way, the image information of the same objects collected from different perspectives can be spatially aligned, providing more flexibility and accuracy for developing intelligent tools based on PLF.

The process of unifying objects recorded from different viewpoints into the same coordinate system is called image registration. Until recently, numerous image registration tasks with critical purposes such as in the medical domain had to be performed manually. Such registration tasks can be time-consuming and even require professional pre-job training for operators when registering complex images. In recent years, researchers have been working on developing automated registration methods to circumvent the limitations of manual registration. When applying artificial intelligence, deep learning can be used to automatically register images and has proven to be a valid upcoming solution [13].

Deep learning was originally applied to optimize the intermediate process of intensity-based iterative registration. Most of the existing research aims at learning a new similarity metric that can be inserted into a classical iterative registration framework [14,15]. However, the improvement of such approaches in multi-modal registration tasks is more evident than in single-modal registration tasks [16]. Moreover, the registration method based on iterative techniques is difficult to apply to real-time registration [17,18]. Therefore, researchers have developed several supervised methods to obtain optimal registration results in one step. Chee et al. [19] proposed a method for predicting rigid transformation parameters without similarity metrics. The samples used to train the model were synthesized from unlabelled data. Experiments on magnetic resonance images of the axial view of the human brain showed that their method outperformed MI optimization-based registration. Rohe et al. [20] used a U-net-inspired CNN [21] to evaluate the displacement field. The SSD between the registered image and the ground-truth image was considered the optimization objective of the network. The authors validated the proposed method on 3D brain MR volumes. The results showed that in uni-modal conditions, the proposed method outperformed LCC Demons-based registration [22].

Supervised learning-based approaches allow for fast registration across applications. However, these labels are usually challenging to obtain and the quality of the registrations using this framework relies on the quality of the ground-truth registrations. These challenges motivate the development of partially supervised or unsupervised approaches. De Vos et al. [23] proposed a multi-stage, multi-scale, mono-modal registration method. The authors used a CNN to perform an affine transformation and subsequently a coarse-to-fine transformation. They validated their method on the Cardiac Cine MRI and Chest CT datasets, where the results also showed that their method was superior to the Elasix toolbox [24]. Hering et al. [25] proposed a weakly supervised transformation estimation. The authors considered both edge-based normalized gradient fields distance and segmentation overlap in the loss function. The experimental results on 2D Cine-MR Images showed that the proposed method outperformed a multi-level registration approach mentioned in [26].

Although unsupervised image-similarity-based registration avoids the need for expert labels of any kind and outperforms conventional iterative techniques [27], the image-similarity metrics (ISMs) used to construct the loss function only evaluate the similarity between the image intensity fields. These similarity quantifications are almost meaningless without context constraints on sampling since they do not indicate the actual measurement error [28]. In other words, these networks are unaware of the displacement errors before and after the registration during training. Rohlfing et al. [29] elaborated on the limitations of using image-similarity metrics and region overlaps in the deformable registration tasks. They also proved that the quality of the registration result is not necessarily related to the magnitude of the quantized value. Although tedious and time-consuming, placing landmarks is still the gold standard for evaluating the registration results.

In this paper, the research objective is to improve visual monitoring of animals such that their behaviour can be identified at the group or individual level, which will enable the farmers to take action for social behaviour so that animals are not lost by accidents or disease. To this end, we propose a fine-tuned semi-supervised registration framework for spatial fusion in a double-camera system. It will fully utilize the complementary information between cameras to improve the performance of multi-object detection, multi-object tracking, and behaviour recognition. Specifically, a small number of manual landmarks are placed to serve as a control signal and provide global constraints during optimization. We validate this approach with pig datasets, which have been captured at real farms. The proposed framework uses a convolutional neural network for mapping all pixels from a moving image to a fixed image by sharing the same parameters for a set of image pairs. The procedure learns a common representation that can align any unseen frame pair from the same recording settings. Aligning a new image pair is achieved by simply applying the learned function made from the given pairs, which results in a fast registration of the new unseen image pair.

This article is organized as follows. Section 2 introduces the image dataset, preprocessing methods, deformation models, evaluation methods, and experimental details. Section 3 provides qualitative, quantitative, and running time evaluations. Section 4 discusses the experimental results, application scenarios, and future improvements.

## 2. Materials and Methods

This section first introduces the dataset used to train and test the proposed methods for estimating spatial correspondence between images from different viewpoints. The section also presents camera calibration, masking, grey-scale conversion, and global initial registration methods to preprocess the raw data. After these steps, the unsupervised and semi-supervised learning-based deformable models are elaborated. Finally, the evaluation method and the implementation details are described.

### 2.1. Dataset Description

**Figure 1:**
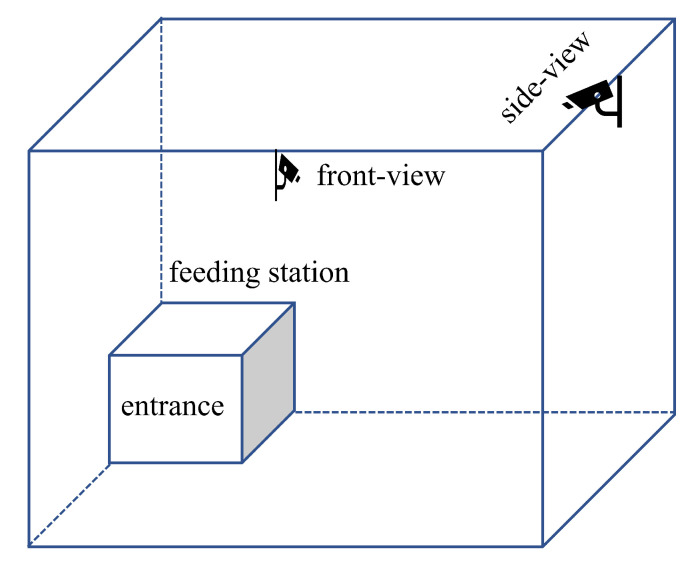
Pig pen layout and lens positions.

**Figure 2:**
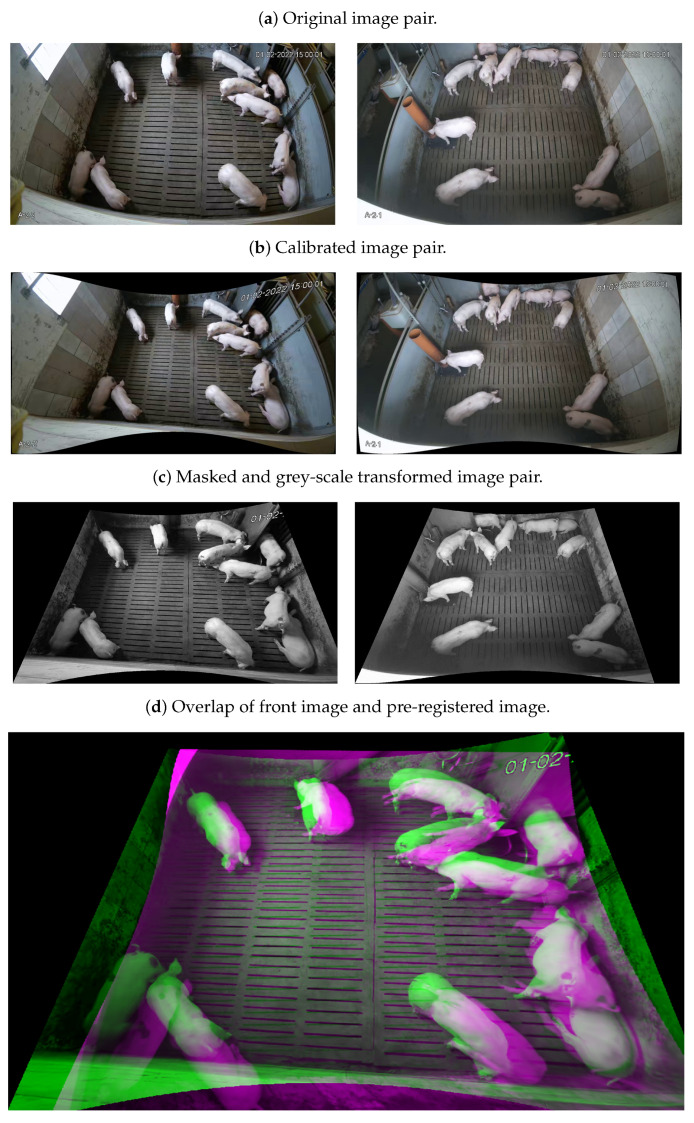
Image pairs at various stages of pre-processing.

A set of video data from pigs is used in this study, recorded at a farm in Volmer, Germany. Each farm enclosure contains 10–11 pigs of the same age and similar body size, where some pigs are artificially coloured on their backs. For our experiment, we have selected video data of 4 pens recorded by 2 cameras (LOREX 4KSDAI168) mounted at the top of the wall opposite the feeding station and at the top of the wall to the left of the feeding station. Figure 1 shows the positions of the cameras in the pig pens. The field of view of both cameras covers the entire living ground and is recorded continuously during the experiment. The data acquisition scenes include daylight changes, solid concrete floors, urine stains, manure, and other sundries. The videos have a resolution of 1280 × 720 pixels and are recorded at 15 fps. The recorded video data is saved as one file every hour. Specifically, we have selected video recordings from 2:00–3:00 p.m. for 7 days in February 2022 (with intervals of 4 days), and then resampled them to video frames at 12-second intervals for experimental material. Due to the smaller pen width, neighbouring pens are visible in the videos of the current pen. Examples of the collected raw image data of the pig pens are shown in Figure 2a.

### 2.2. Data Pre-Processing

#### 2.2.1. Camera Calibration

The process of evaluating camera parameters using images containing a specific calibration pattern is called calibration. When these parameters are obtained, the distortion from the image can be removed or the location of the camera can be predicted. To obtain these parameters, the calibration pattern (usually a checkerboard) inside the real-world image should be captured. For the calibration, we customize a checkerboard of (1 × 1 sq. meter) and position it at different angles in front of each of the two lenses to obtain the camera parameters. Since the lens and image plane of the camera are parallel in orientation, we only focus on the correction of radial distortion. Therefore, we omit the camera parameters and directly describe the distortion transformation. The distorted points are denoted as $\left(x_{\mathrm{distorted}},y_{\mathrm{distorted}}\right)$:

(1) $$\begin{array}{c} x_{\mathrm{distorted}}=x\left(1+k_{1}\cdot r^{2}+k_{2}\cdot r^{4}+k_{3}\cdot r^{6}\right),\\ y_{\mathrm{distorted}}=y\left(1+k_{1}\cdot r^{2}+k_{2}\cdot r^{4}+k_{3}\cdot r^{6}\right), \end{array}$$

where *x* and *y* are normalized image coordinates; $k_{1}$, $k_{2}$, and $k_{3}$ are radial distortion coefficients of the lens with $r^{2}=x^{2}+y^{2}$. In this part, only $k_{1}$ and $k_{2}$ have been used for calibration. The calibrated video frames are shown in Figure 2b.

#### 2.2.2. Masking and Grey-Scale Conversion

Since this study focuses on the pixel-level correspondence between side-view video frames and front-view video frames in the actual pig pen, masking the content outside the pen in video frames allows the algorithm to pay more attention to the actual transformation. RGB images contain three channels capable of recording rich color information. However, in real farming scenes, the lighting conditions change frequently, which has a direct impact on the generalization of the model [30]. An effective way is to convert RGB images into grey-scale images [31], so that the learning ability of the model can be centralized in the tasks of the interest. Figure 2c illustrates the masked and grey-scaled image pair.

#### 2.2.3. Baseline Method

Projective transformation is considered the baseline method in this work. For each pen, 35 landmark pairs are placed on a randomly chosen frame pair to obtain the parameters for projective transformation. Then these parameters are used to align the remaining image pairs in the same pen. Figure 2d shows the outcome after overlapping a pre-registered side-view frame (magenta) with its corresponding front-view frame (green). Landmark pairs used to perform pre-registration are not employed for training and testing.

### 2.3. Deformation Models

The deformation model is refining the pre-registration transformation to a full registration of higher quality. We assume that $I_{f}:\Omega_{f}\to R_{n}$ and $I_{m}:\Omega_{m}\to R_{n}$ denote the front-view images (subscript *f*) and the corresponding pre-registered side-view images (subscript *m*), respectively, where in our settings n=2. The problem of finding a dense non-linear transformation is reduced to an optimization problem, specified by

(2) $$arg minE\left(\Phi \right)=\min_{\Phi }\left(M(I_{f},I_{m}\left(\Phi \right))+\mu R\left(\Phi \right)\right),$$

where $I_{m}\left(\Phi \right)$ is $I_{m}$ warped by Φ, the function *M* measures the similarity between the fixed image $I_{f}$ and the moving image $I_{m}$ that has been transformed by Φ. Function R(Φ) represents a constraint imposed on the deformation field Φ, ensuring that the deformation field composed of displacement vectors of all pixels is smooth.

**Figure 3:**
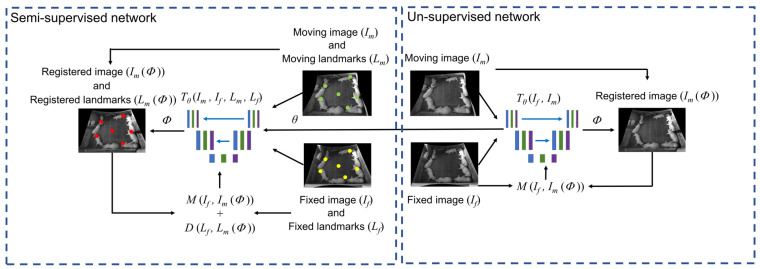
The proposed method consists of two stages. First, train the unsupervised network with image pairs without landmarks to obtain the weights across pig pens and over time; second, import these weights into the semi-supervised network and fine-tune with samples labelled with landmarks.

Figure 3 presents an overview of the proposed method. First, an unsupervised convolutional neural network is designed to parameterize the mapping function $T_{\theta }\left(I_{m},I_{f}\right)=\Phi$ using unlabelled training samples. After this, a semi-supervised network is constructed, which has the same convolutional layers as the unsupervised model so that it can adopt the learned weights from the unsupervised network. Besides the similarity metric *M* between $I_{f}$ and $I_{m}\left(\Phi \right)$, the loss function of the semi-supervised network also includes an item that refers to the distance errors between transformed landmarks and the ground-truth landmarks. Finally, a small number of annotated samples are fed into the semi-supervised network to fine-tune the pre-trained weights.

This design is motivated by following the concept of transfer learning. In this experiment, the task of registering image pairs with landmarks can be regarded as the target domain; the task of registering image pairs without landmarks can be seen as the source domain. Since landmark annotation is labour-intensive and time-consuming, the cost of learning directly from the target domain from scratch is too high. Fortunately, there are large available unlabelled samples that can be used for source-domain learning, so that the knowledge learned from the source domain can be exploited to assist in learning new knowledge in the target domain.

#### 2.3.1. Unsupervised Deformation Model

In this part, the network *T* is modelled using a set of learnable parameters θ. The architecture of the network is inspired by a U-Net, which is similarly composed of encoder, decoder, and skip connections. This is motivated as follows.

The architecture of the proposed network is adopted from the U-Net, since the encoder–decoder structure shows state-of-the-art performance on semantic segmentation tasks, which meets the requirement to learn displacement vectors at the pixel level. Moreover, U-Net can be trained end-to-end with a very small number of samples. Finally, the up-sampling layer fuses the features of its corresponding down-sampling layer through the skip connection, which can provide more spatial domain information for image registration.

**Figure 4:**
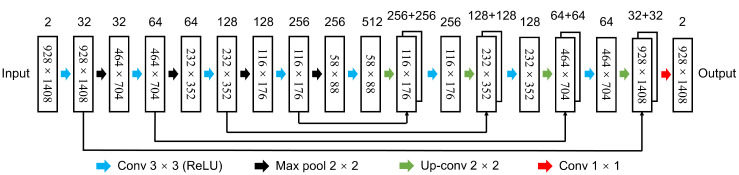
The architecture of the proposed network.

Figure 4 depicts the architecture of the proposed network; Table 1 and Table 2 show the specifications of the convolution filters used in up-sampling and down-sampling.

The proposed network combines the moving (side view) and the fixed images (front view) as a single input with a size of 928 × 1408 × 2 pixels. The kernel size of all convolutional layers is 3 × 3, and Leaky ReLU is used as the activation function. In the encoding, the convolutional kernels capture the hierarchical features of the images at different resolution scales. The 2 × 2 pooling layers halve the size of the feature maps calculated by the previous convolutional layers until the smallest layer is reached. The feature maps also stream to the decoder and combine with the deconvolution output, which is critical for the estimation of the deformation field. The size of the receptive field of the last convolutional layer determines the maximum moving distance for the pixels. In the decoding, the deconvolution layers double the size of the feature maps to match the counterpart from the encoder. A convolutional layer with two 1 × 1 kernels compose the last layer of the decoding. The output Φ is of size 928 × 1408 × 2, which refers to the moving distance in the spatial directions for each pixel. In each iteration, the mapping from $I_{m}$ to the deformed $I_{m}\left(T_{\theta }\left(I_{m},I_{f}\right)\right)$ is implemented by a spatial transformer network [32]. This module enables the spatial transformation within the network and requires no modification to the minimizing optimization process on E(Φ) in Equation (2). Furthermore, the differentiable characteristic of the module makes it suitable to be used in gradient descent-based networks. Finally, for robustness to intensity variations, the mean-squared error (MSE) is used to measure the similarity between $I_{m}$ and $I_{f}$. The MSE is specified as

(3) $$\mathrm{MSE}=\frac{1}{n}\sum_{i=1}^{n}{\left(\;I_{f(i)}-I_{m(i)}\left(\Phi \right)\;\right)}^{2},$$

where $I_{f(i)}$ is the intensity value of individual pixels *i* (with *n* the number of pixels) in $I_{f}$ and $I_{m(i)}\left(\Phi \right)$ indicates the intensity value of pixels in $I_{m}$. We adopt from [33] a regularization process, where diffusion regularization is used to regularize the spatial gradients given by

(4) $$R\left(\Phi \right)=\sum_{(x,y)\in \Omega }{∥\nabla \Phi \left(x,y\right)∥}^{2}.$$

The optimal solution to the spatial transformation is searched by minimizing the loss function:

(5) $$\begin{array}{ccc} \; & L(I_{f},I_{m},\Phi \left(\theta \right))=\; & \mathrm{MSE}\left(I_{f},I_{m}\left(\Phi \left(\theta \right)\right)\right)+\lambda \sum_{(x,y)\in \Omega }{∥\nabla \Phi \left(x,y\right)∥}^{2}, \end{array}$$

where λ is the regularization parameter.

#### 2.3.2. Semi-Supervised Deformation Model

Besides image pairs, there is a set of corresponding landmark points in the input in the semi-supervised network. The configurations of encoder, decoder, and skip connections in the unsupervised network are adopted here with some adaptations. Specifically, in the semi-supervised framework, there is an additional spatial transformation layer that is expected to transform the landmark points $\left(x_{lp},y_{lp}\right)$ on $I_{m}$ using the intermediate deformation field:

(6) $$\left(x_{lp,m}^{\prime },y_{lp,m}^{\prime }\right)=f_{stl}\left(x_{lp,m},y_{lp,m}\right).$$

Here, $\left(x_{lp,m},y_{lp,m}\right)$ are the coordinates of the landmarks on $I_{m}$ and $f_{stl}$ is the spatial transformation function related to θ that transform the landmarks to the supposed position. Since we aim at minimizing the positioning errors of the landmarks, an extra loss term is added to the loss function of Equation (5). To this end, after obtaining the transformed landmarks in each iteration, the mean value of the Euclidean distances between transformed and ground-truth landmarks is calculated and add it to the loss function. Finally, the learning process minimizes the modified extended loss function:

(7) $$L\left(I_{f},I_{m},\Phi \left(\theta \right)\right)+\mu \frac{1}{k}\sum_{j=1}^{k}{\left[\left(x_{lp,f},y_{lp,f}\right)-\left(x_{lp,m}^{\prime },y_{lp,m}^{\prime }\right)\right]}^{2},$$

where μ is a hyper-parameter and *k* is the number of landmark pairs.

### 2.4. Evaluation Method

To validate the proposed methods, six landmarks are manually selected on each moving and fixed image in the test set and are used as ground-truth data to measure the overall misalignment for registration. The registration accuracy can be estimated by the target registration error (TRE), which indicates the average squared Euclidean distance between the fixed, chosen landmarks and their matching correspondences in the deformed images (moving landmarks). The TRE is specified as follows:

(8) $$\mathrm{TRE}=\frac{1}{k}\sum_{i=1}^{k}{\left(\;\left(x_{lp,f},y_{lp,f}\right)-\left(x_{lp,m}^{\prime },y_{lp,m}^{\prime }\right)\;\right)}^{2}.$$

**Figure 5:**
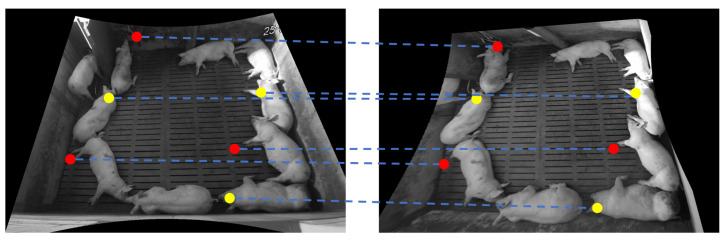
Examples of two types of landmarks and their correspondences. Red dots indicate background objects and yellow dots indicate the foreground.

Furthermore, we have annotated two types of landmarks in the images. The first landmark type is located on foreground objects in the images (e.g., the yellow points in Figure 5). The second landmark type is located on background objects in the scene (e.g., the red points in Figure 5). Both types are important because aligning foreground objects while maintaining the relative positions between foreground and background objects is a prerequisite for many object tracking applications.

### 2.5. Implementation Details

The code is implemented using Tensorflow 2.6.0. The network *T* is a U-Net-based encoder–decoder network with residual connections [34]. The instance normalization layer is used in all skip connections [35] and Kaiming [36] initialization is applied for all networks. The experiments are conducted on a single GeForce RTX 2080Ti. An Adam Optimizer [37] is used on a minibatch of size 4 with the learning rate $l_{r}=1\times 10^{-4}$. The model is trained for 200 epochs and linear learning-rate decay is activated after 100 epochs. Separate networks are trained with different λ and μ regularization values until convergence occurs and we report results on the test set, which is separated and different from the training set.

### 2.6. Experiments

The proposed method focuses on computing a registration field across pens and over time. The dataset contains 6291 image pairs, including 2087 image pairs in each of the A1, A2, and A3 pens and 30 image pairs of the A4 pen. Each image pair consist of two 24-bit RGB video frames captured by high-resolution sensors. Prior to the global alignment phase, images are calibrated, masked, and grey-scaled to the resulting input frames with a size of 928 × 1408 pixels. The initial image registration is based on the projective transformation, with 35 manually placed landmarks, also considered as the baseline for the experiments. However, some non-linear misalignment in the dataset also occurs, which cannot be handled by the initial (rigid) registration. For each of the A1, A2, and A3 pens, we randomly split the data into 2057, 20, and 10 image pairs for training, fine-tuning, and testing, respectively. In order to verify the generalization ability of the models on registering image pairs from a new pen, the image pairs of A4 are only used for testing. In 3 fine-tuning sets and 4 testing sets, 6 landmark pairs are placed in the image pairs: 3 landmark pairs are on the animals, while the other 3 are located in the background.

When training the unsupervised network, 6171 non-annotated training image pairs from A1, A2, and A3 are supplied to the network. Each input pair consists of a fixed image and a moving image. Whereas the semi-supervised network is only trained using annotated samples of the fine-tuning sets, the input of the semi-supervised network has an additional layer for landmark coordinates in the moving images. In the fine-tuning session, weights obtained after unsupervised learning, which are able to extract general features across pig pens and distributions, are adopted and inserted into the semi-supervised framework. The TRE definition in Equation (8) of the test image pairs is used to evaluate the performance of the models on the seen and unseen pens. The registration accuracy is improved by regularizing the optimization process using the actual average squared misalignment distance represented by the TRE.

## 3. Results

### 3.1. Quantitative Evaluation

We have compared the average registration error in terms of TRE for different registration networks with the baseline method of only global projective transformation in Table 3. Specifically, in addition to the mean-squared error (MSE), the other two commonly used loss terms are also involved, i.e., normalized cross-correlation (NCC) and the sum of squared differences (SSD). These commonly used loss terms NCC and SSD are inserted into the training optimization by maximizing and minimizing, respectively. We also compare the involved networks with feature-based and intensity-based approaches at the bottom of the Table 3. All methods are evaluated on the same test sets. The first baseline method considered is speeded-up robot features (SURF) [38]. It is invariant to changes in scale, rotation, and illumination and has proven to be much faster than the scale invariant feature transform (SIFT) [39]. Registration is based on matching features represented by these descriptors. First, SURF descriptors are extracted from the calibrated, resized, and masked RGB image pairs. Then, these features are matched and estimated with the 2D geometric transformation for matching point pairs, using an affine transformation. On this basis, we also apply a non-rigid transformation, specifying a pyramid vector [100, 50, 25] for iterative computation. A Gaussian smoothing filter is used to harmonize the accumulated field at each iteration. Deformation fields are calculated through a non-parametric diffeomorphic image registration algorithm [40] and re-sampled using linear interpolation. The second method is the mono-modal intensity-based approach. The method uses affine transformation for global alignment. Gradient magnitude tolerance, relaxation factor, and maximum iterations are set to $1\times 10^{-4}$, 200, and 0.5, respectively. The mean-squared error is used in the loss function to control the minimization of the total error to optimize the similarity of the images. For the baseline methods, the post-processing method for nonlinear transformation is the same as used in the previous feature-based method.

As can be observed from Table 3, all networks achieve better results than the baseline methods, except for the semi-supervised network. Two values are reported, one for the measured error on the background (left) and the second based on foreground annotations (right). The SURF-based method performs comparable to the mono-modal intensity-based method on average TRE, and both methods are 3.02 and 2.53 pixels lower in average TRE than the projective transformation. They also have comparable TRE values on foreground and background landmarks. The unsupervised model trained with MSE outperforms the networks trained with NCC and SSD. On one hand, it has a lower average TRE value (33.31 pixels). On the other hand, it has a lower error on both the task of aligning foreground animals and aligning background. The table also shows that a fine-tuned network using MSE as the loss term can reduce the average TRE from 40.12 pixels to 22.95 pixels, yielding the most significant improvement over other frameworks, while the TRE metrics of the foreground and background registration are lower than that of the unsupervised network. However, the semi-supervised network leads to degraded forms of registration. The semi-supervised networks achieve the most significant registration error compared with the unsupervised networks and the fine-tuned networks when the same loss term is used.

**Figure 6:**
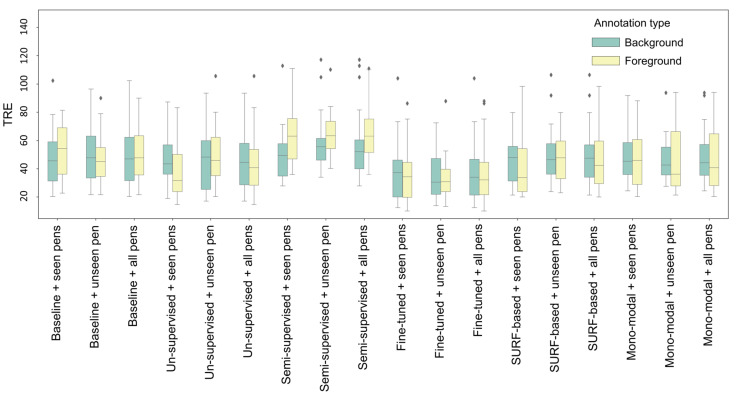
Boxplots of TRE scores of each image registration method for aligning the background and the foreground objects. For each method a cluster of three results are grouped: seen pens, unseen pen, and all pens.

The distributions of the TRE score for each method are visualized as box plots. For comparison purposes, the foreground and background registration performances of each technique on three different test sets (seen pens, unseen pens, and all pens) are shown in Figure 6. As can be observed, the SURF feature-based and mono-modal intensity-based methods achieve comparable TRE measures to the baseline on seen-pen and unseen-pen test sets. Both perform slightly better than the baseline on the test sets in all pens. Models based on unsupervised learning and fine-tuning strategies correct more errors in registration on the seen-pen test sets than on the unseen-pen test set. The fine-tuned training network gives the best registration accuracy on each test set. It performs better in terms of low TRE when aligning the background than when aligning the foreground.

Since the set size for testing is less than 2000, the Shapiro–Wilk test results show that only the TRE values calculated from the image pairs registered by the unsupervised and the fine-tuned network on the test set of unseen pens conform to the normal distribution (*p* > 0.05). We use the Wilcoxon sign rank test and T-test to evaluate whether these methods are significantly improved compared to the baseline method. SURF feature-based and mono-modal intensity-based methods show no significant improvement over the projective method on the test sets (*p* > 0.05). In contrast, both the unsupervised and the fine-tuned methods have significant differences over the projective method on the test sets (*p* < 0.05). Furthermore, the TRE values of the fine-tuned model are significantly lower than the numbers of the unsupervised model on the three test sets (*p* > 0.05).

### 3.2. Qualitative Evaluation

**Figure 7:**
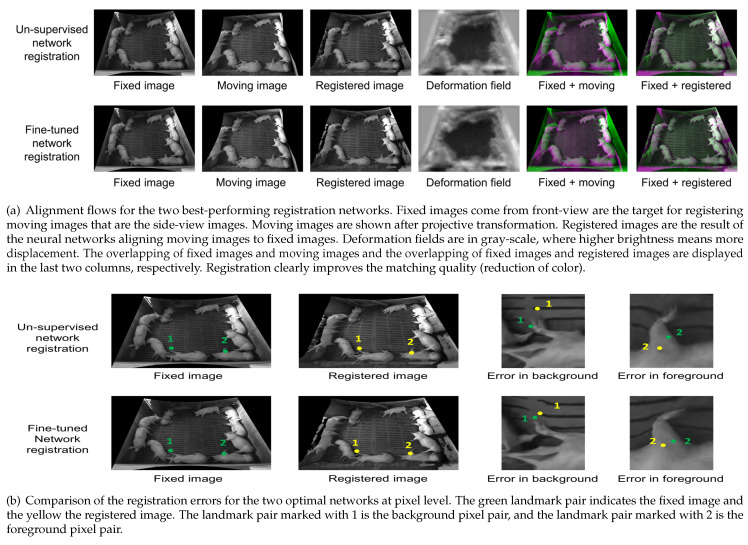
Illustrations of non-linear dense registration effects and errors of un-supervised and fine-tuned networks in surveillance of a pen with pigs.

Figure 7a shows that the proposed registration networks successfully align image pairs from real pig pens. In the first row of Figure 7a, the unsupervised method aligns a pair of images. As seen from the visualization of the deformation field, minor deformation is applied to the ground area of the current pen (see the black centre in the deformation field). At the same time, most deformation is applied to the body of the pigs and visual field boundaries. Furthermore, in the second row in Figure 7a, the fine-tuned network is used to deform the same moving image. The deformation field calculated by the fine-tuned network is similar to the field of the first row. Although it is similar, it will result in a better registration alignment. To visualize the full alignment within this image pair, we overlay (with semi-transparency) the whole scene of the fixed image on top of both moving images before and after the deformable registrations. Grey regions in the composite image show where the two images have the same intensities. Magenta and green regions show where the intensities are originating from moving and fixed images, respectively. The overlay of fixed and registered images shows the most grey-scale values, highlighting the higher quality of both images.

Figure 7b shows the transformation results using deformable fields for landmarks. Two landmark pairs are visualized on a fixed image and its corresponding registered image. The landmark pair that relates to the background is denoted as 1, the landmark pair that is a key point of the pigs is marked as 2. Landmarks on the fixed image are filled with green while landmarks on the registered image are yellow. In the first row in Figure 7b, we use the deformation field calculated by the unsupervised network to transform the landmarks, while in the second row in Figure 7b, the same image pair and the same landmark pairs are used to show the alignment results of the fine-tuned network. As can be observed, the transformation from the fine-tuned network brings the corresponding landmarks closer.

**Figure 8:**
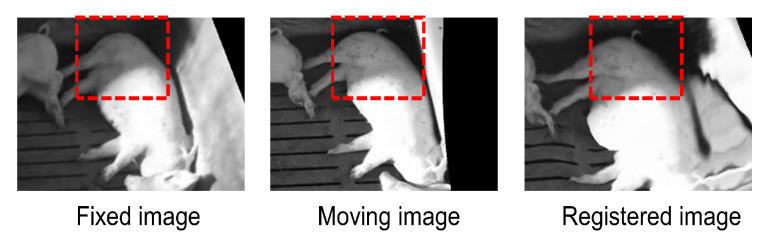
The proposed method retains the appearance features specific to moving images while aligning moving images to fixed images.

A final check is obtained on the visual quality of the registration. It is important that the skin or other details of the animals are preserved after registration. Therefore, an example is shown here. As can be observed from Figure 8, the pigtail is not visible in the fixed image, while the moving image captures it. After registration, the pigtail is preserved in the registered image. Spatial alignment of image content while maintaining unique appearance features provides a good starting point for subsequent tasks such as object detection, tracking, and behaviour recognition.

### 3.3. Execution Time Analysis

Table 4 presents the execution time results using an i7- 7700K CPU core and a GeForce RTX-2080Ti GPU. The elapsed execution time is measured for computations following the projective alignment (pre-registering step), which is shared by all presented methods. To our knowledge, SURF-based and mono-modal intensity-based registrations have no complete implementation for GPU-based execution. The SURF method has a registration time of 8 s on the CPU, while the mono-modal intensity-based approach requires about 28 s on the CPU. The presented learning-based methods are more than 10 times faster in execution on a GPU than on a CPU for the registration task. When registering a new image pair on GPU, the time consumption is limited to 0.5 s.

## 4. Conclusions and Discussion

This work proposes a semi-supervised, fine-tuned learning algorithm that can be used to register double-camera videos for animal surveillance in real farming. Considering the complexity and the time investment for annotation, the proposed method only requires a few image pairs with landmarks to obtain an accurate deformation field. Fine-tuning is obtained as follows. First, an unsupervised network is trained with unlabelled data. Then the network weights are adopted to the semi-supervised network, after which the semi-supervised network is fine-tuned with training data using annotated images. The fine-tuned network has demonstrated an average registration error (TRE value) of 22.95 pixels, which is 17.17, 10.36, 22.72, 14.02, and 14.51 pixels lower than the baseline, unsupervised, semi-supervised, SURF feature-based, and mono-modal intensity-based methods, respectively. The above results show that the knowledge learned in the unsupervised registration process can be well transferred to the semi-supervised learning framework, and no negative transfer occurs.

One of the most important tasks in animal surveillance is to track moving objects independently, using videos from the double-camera system. In this dataset, pigs are about 240 ± 37 pixels long and 125 ± 41 pixels wide. Since the average TRE is about 22 pixels in the proposed method, the deformation field is sufficient to move the appearance pixels of the same object from two viewpoints into the same or adjacent receptive field in the tracking network.

The statistical result shows that the unsupervised and fine-tuned networks can significantly reduce the TRE compared to the baseline on all test sets. Furthermore, a statistically significant improvement has also been detected in the fine-tuned network over the unsupervised network on the unseen test set using the T-test. On one hand, the slightly higher foreground TRE value is due to the lack of depth information. On the other hand, the foreground misalignment error is already more significant than the background errors after pre-registration.

The proposed method can register the targets recorded using different camera positions into the same coordinate system. In other words, by overlapping the registered image pairs together, image information from two perspectives can be presented in the target space region, which is helpful for multi-object tracking, re-identification, and behaviour-recognition tasks. In addition, a more promising point is that it provides a new idea for alleviating the occlusion problem in single-camera systems, because even if an object is completely occluded in a certain view, after registration and fusion, there will be appearance information available from another perspective at the position where it should appear, so that the model can perceive the presence of the same object.

The end-to-end strategy of the network enables the estimation of a deformation field for transforming the entire image, with a displacement vector for each pixel. However, it should be noted that the images registered by the proposed method retain their unique appearance features recorded from the side-view camera. Such features are very friendly to downstream tasks such as re-identification because the proposed method can integrate unique appearance features of objects in the same local space, which is very beneficial for distinguishing different objects for analysis at the individual level.

The semi-supervised model, which learns from scratch, does not show comparable performance in the experiment, because it is based on a limited number of training samples. In the near future, data augmentation techniques should be adopted to expand the variety of the training sample without adding extra manual effort.

The expensive challenge of registering a new image pair has been converted into an optimization problem, aggregated over a set of samples. Following the experimental setup, the model takes 2 days to compute on a single GeForce RTX-2080Ti GPU to obtain the reported parameters. After this, a new image pair can be registered within 0.5 s. The current solution is processing on a frame-by-frame basis. In the future, we may explore whether a common deformation field can be found to register two videos of the same pen and provide online processing.

In the current implementation, the input image pairs need to be pre-aligned to obtain a dense deformation field with good registration performance. A future research topic is constructing a whole learnable registration framework, which involves pre-registration as part of global optimization.

The original U-Net simply concatenates the features of the down-sampling layer directly into the up-sampling layer of the same depth. We can first use the attention module to process the feature maps of the down-sampling layer and the feature maps of the upper layer of the corresponding up-sampling layer and then concatenate them with the current up-sampled feature map. Thereby, the attention of the model will focus on the object region (foreground).

Although the proposed method has been verified to be effective on real data, the models do not emphasize constraints on maintaining geometric properties. Offering regularization to preserve geometric properties is one of the priorities of future work.

## Figures and Tables

**Table 1:** Specifications of the down-sampling convolution filters.

Down-Sampling Layers	1	2	3	4	5
Kernel number	32	64	128	256	512
Kernel size	3×3	3×3	3×3	3×3	3×3
Activation function	ReLU	ReLU	ReLU	ReLU	ReLU
Padding	Same padding	Same padding	Same padding	Same padding	Same padding
Stride ($s_{h}$, $s_{v}$)	(1, 1)	(1, 1)	(1, 1)	(1, 1)	(1, 1)
Pooling	(2, 2)	(2, 2)	(2, 2)	(2, 2)	(2, 2)

**Table 2:** Specifications of the up-sampling convolution filters.

Up-Sampling Layers	1	2	3	4	5
Up-sampling (deconvolution)	2×2	2×2	2×2	2×2	-
Kernel number (deconvolution)	256	128	64	32	-
Kernel size (deconvolution)	2×2	2×2	2×2	2×2	-
Activation function (deconvolution)	ReLU	ReLU	ReLU	ReLU	-
Concatenate	Yes	Yes	Yes	Yes	-
Kernel number (convolution)	256	128	64	32	2
Kernel size (convolution)	3×3	3×3	3×3	3×3	1×1
Activation function (convolution)	ReLU	ReLU	ReLU	ReLU	ReLU
Padding (convolution)	Same padding	Same padding	Same padding	Same padding	No padding
Stride ($s_{h}$, $s_{v}$) (convolution)	(1, 1)	(1, 1)	(1, 1)	(1, 1)	(1, 1)

**Table 3:** Measured TRE values for the proposed registration networks and baseline registration methods (NCC: normalized cross-correlation; SSD: sum of squared differences; left metric number: background; right metric number: foreground).

Methods	MSE	NCC	SSD	Optimal TRE	Avg. TRE
Projective transformation (proposed)	-	-	-	36.01/44.22	40.12
Unsupervised learning network	30.92/35.70	32.24/36.66	33.48/38.96	30.92/35.70	33.31
Semi-supervised learning network	48.46/47.89	39.13/52.22	41.06/57.29	39.13/52.22	45.67
Fine-tuned learning network	20.55/25.36	25.77/28.67	22.08/28.30	20.55/25.36	22.95
Feature-based registration (SURF)	-	-	-	38.09/35.86	36.98
Mono-modal intensity-based registration	35.11/39.81	-	-	35.11/39.81	37.46

**Table 4:** Average execution times (and std. devs.) on CPU and GPU for the proposed registration networks and baseline methods.

Methods	CPU	GPU
Unsupervised learning network	1.52 (2.26)	0.12 (0.13)
Semi-supervised learning network	1.48 (4.18)	0.10 (0.06)
Fine-tuned learning network	1.51 (2.77)	0.13 (0.17)
SURF-based registration	8.20 (1.36)	-
Mono-modal intensity-based registration	28.51 (19.65)	-

## Data Availability

Not applicable.

## Supplementary Materials

The following supporting information can be downloaded at: https://www.mdpi.com/article/10.3390/foods12010084/s1.
