# Supplementary material for: Double-Camera Fusion System for Animal-Position Awareness in Farming Pens
Source: Foods. 2022 Dec 23;12(1):84. doi: 10.3390/foods12010084 (PMC9818956; doi:10.3390/foods12010084)
Supplement: Supplementary file 1 [file foods-12-00084-s001.zip › foods-2006127-supplementary.pdf]

WAGENINGEN UNIVERSITY
WAGENINGEN UR

For quality of life

Memo

Corporate Education
and Research

DATE
December 23, 2021,

POSTAL ADDRESS
Bode 120
The Netherlands

VISITORS' ADDRESS
Bornse Weilanden 5
6708 WG Wageningen

INTERNET
www.wageningenuniversity.nl

HANDLED BY
Rob Steenmans

TELEPHONE
+31 (0)317 484206

EMAIL
rob.steenmans@wur.nl

TO
Liesbeth Bolhuis

FROM
Rob Steenmans
Animal Welfare Officer

Dear Liesbeth Bolhuis,

The Animal Welfare Officer assessed the work protocol**: IMAGEN project - Social behaviour modelling in pigs,** received on December 21, 2021.

It is the opinion of the AWO that this is not an animal experiment as referred to in the Dutch Act on Animal Experiments, since the experimental procedures described in present protocol will cause less pain or distress than the insertion of a needle under good veterinary practice.

Yours sincerely,

Rob Steenmans
Animal Welfare Officer

Wageningen UR (Wageningen University and various research institutes) is specialised in the domain of healthy food and living environment.
